# Stiff-Person Syndrome: A Case Report and Review of the Literature

**DOI:** 10.22599/bioj.130

**Published:** 2019-04-16

**Authors:** Joe Smith, Hayley Storey

**Affiliations:** 1 Manchester Royal Eye Hospital, GB

**Keywords:** stiff-person syndrome, anti-glutamic acid decarboxylase antibodies, vertical diplopia, muscle spasms, skew deviation, nystagmus

## Abstract

**Aim:**

To report a case of sudden onset vertical diplopia, blurred vision, and muscle spasms.

**Methods:**

This is a case report of a 57-year-old female who presented to the accident and emergency department with a one day history of vertical diplopia and a two week history of lower limb spasticity secondary to muscle spasms.

**Results:**

The patient had no significant medical or ocular history. Orthoptic investigation initially revealed a left inferior rectus (IR) underaction. Possible diagnoses at this point were thought to be isolated IR weakness, myasthenia gravis or skew deviation. An urgent MRI scan was arranged and blood tests were taken. MRI showed no abnormalities. Blood tests were normal, however, the acetylcholine receptor antibody serum test (ACH-R) was 0.43 nmol/L, which is at the high end of normal. At the follow-up visit, the IR weakness had deteriorated and the patient had also developed gaze-evoked nystagmus. An appointment with the neurologist and neuro-ophthalmologist was expedited. When the patient returned, she reported that her neurologist had diagnosed her with stiff-person syndrome (SPS). The patient had also developed a laterally alternating skew deviation and reported that she had undergone a course of intravenous immunoglobulin (IVIG). The patient was prescribed diazepam and gabapentin. Due to the lack of recovery, persistent diplopia and oscillopsia, monthly IVIG have been prescribed.

**Conclusion:**

There is currently a paucity of literature relating to ophthalmic problems with SPS and how they are best treated. Previous reports have established that there is a link with myasthenia gravis, with many patients going on to develop myasthenia. Treatment of SPS is lacking large evidence-based studies. However, treatment with muscle relaxants and anticonvulsants has provided a good outcome for some. Further research is required to develop an evidence-based approach to treating the ophthalmological problems patients with SPS experience. This case report highlights the importance and value of orthoptists in investigating and monitoring patients with stiff-person syndrome.

## Introduction

Stiff-person syndrome (SPS) is a rare neurological disorder which affects the nervous system and affects less than one million people worldwide (Brown & Marsden 1999; Hadavi et al. 2011; Lorish, Thorsteinsson & Howard 1989). SPS is rather unique as it lacks significant similarity to any other neurological disorder, however, it has the features of an autoimmune disorder as the majority of patients have glutamic acid decarboxylase (GAD) antibodies (Brown & Marsden 1999; Hadavi et al. 2011; Lorish, Thorsteinsson & Howard 1989). The cause of SPS is relatively unclear (Dalakas 2008, Hadavi et al. 2011; Lorish, Thorsteinsson & Howard 1989).

SPS is characterised by progressive rigidity and stiffness, accompanied by muscle spasms, commonly affecting the trunk and/or limbs (Brown & Marsden 1999, Dalakas 2008, Hadavi et al. 2011; Lorish, Thorsteinsson & Howard 1989). The symptoms are progressive and may fluctuate (Dalakas 2008). We describe a patient who presented with complaints of muscles spasms and vertical diplopia that was subsequently found to have SPS.

## Case report

### Presentation

A 57-year-old female presented to the accident and emergency department with a two week history of muscle spasms and a one day history of intermittent diplopia. She reported that due to the muscle spasms she was struggling to walk and climb stairs. Due to intermittent diplopia, she was referred to the emergency eye department and was seen by an orthoptist.

The case history at the first assessment revealed that the patient had been on holiday two weeks earlier when she started to experience muscle spasm and gait problems. When the patient returned home, she reported that she began to notice binocular vertical diplopia on laevoversion. After the onset, the patient reported no change or worsening of diplopia. The patient also reported blurred vision for the past several months and a feeling that her ocular status was not normal; her last refraction had been seven months prior. There was no significant ocular history other than glasses wear since adulthood, no obvious precipitating factors and no compensatory head posture. Past medical history was normal and there was a family history of diabetes.

**Figure 1:**
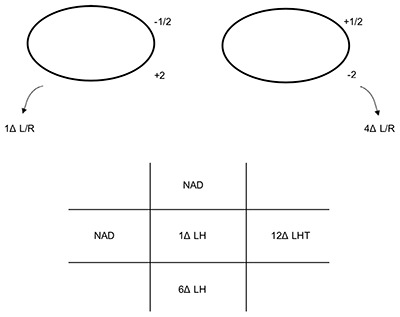
The patient’s ocular movements and prism cover test measurements (at 3 m) at presentation. Note: the arrows denote the BHTT measurements. LH: left hyperphoria, LHT: left hypertropia.

**Figure 2:**
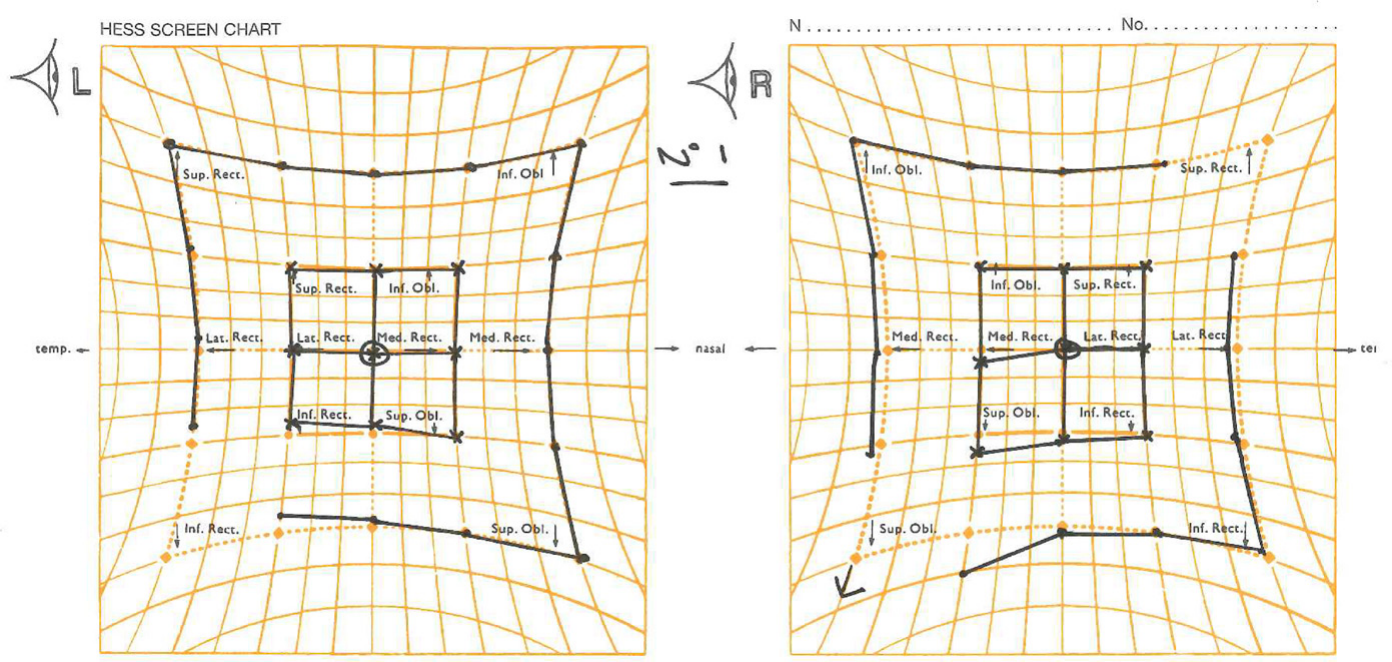
Hess chart from visit 1. Showing left inferior rectus underaction and right superior oblique overaction.

Visual acuity (VA) was right eye 0.0, left eye 0.04 LogMAR acuity. On cover test (CT) a left hyperphoria with good recovery was noted for both near and distance. Binocular single vision (BSV) was present at both near and in the distance. At near, the deviation measured 2Δ LH. Ocular movements (OM) and the prism cover test (PCT) measurements at 3 m are shown in Figure 1. No nystagmus was seen when testing ocular movements and diplopia was reported on laevoversion and laevodepression. There were no signs of myasthenia gravis (e.g. variability) and clinical tests were negative. The Bielschowsky head tilt test (BHTT) was also negative. A completed Hess Chart is shown in Figure 2. Visual field testing showed no defects. Potential diagnoses at this time were thought to be isolated left inferior rectus palsy (IR), skew deviation or myasthenia gravis. Isolated IR palsies are rare and there were no signs of myasthenia gravis. Skew deviation was thus thought to be the most likely diagnosis in view of the other neurological signs. A referral for blood tests and an MRI (paying particular attention to the brainstem) was suggested to the emergency eye department doctors. The patient was given Blenderm (3M™) occlusion by the orthoptist covering her entire left lens, as she declined sector occlusion.

### Six days after presentation

**Figure 3:**
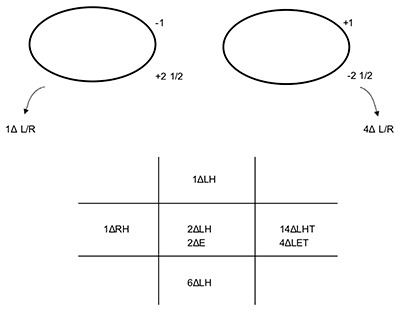
The patient’s ocular movements and prism cover test measurements (at 3 m) six days after presentation. Note: the arrows denote the BHTT measurements. LH: left hyperphoria, E: esophoria, LHT: left hypertropia, LET: left esotropia, RH: right hyperphoria.

**Figure 4:**
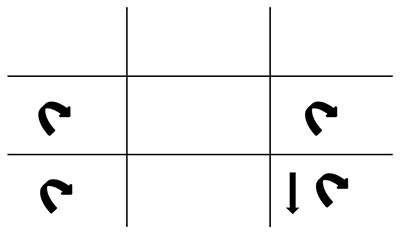
Recording of our patient’s nystagmus. The patient had gaze-evoked rotary nystagmus with medium amplitude and frequency in all demonstrated positions. Our patient also had gaze-evoked downbeat nystagmus on laevo-depression which was only present in the left eye.

The patient returned to the orthoptic department and felt there had been no changes. VA was right eye 0.0, left eye 0.04 LogMAR acuity. On CT it was noted that she had a minimal left hyperphoria with good recovery at near and with diplopia prior to recovery in distance. This measured 1Δ LH at near. The patients OM and PCT at 3 m measurements are shown in Figure 3. The left IR weakness appeared to be worsening and the patient now also appeared to have a right over left deviation on dextro-version, however, no significant muscle underaction could be seen on OM testing. Atypical gaze-evoked nystagmus was now present (Figure 4). Saccades were tested qualitatively and noted to have a normal latency but were slightly hypometric (vertical > horizontal). The upright-supine test was performed and a negative result was obtained. In view of the assessment, the patient’s condition seemed to be worsening. Blood tests had all come back with normal levels, however the acetylcholine receptor antibody serum test (ACH-R) was 0.43 nmol/L (reference range: 0–0.44). This result is at the high end of normal. Thus, it was suspected that this could be a possible diagnosis. However, MuSK antibodies were also tested for with a negative result. The MRI showed only multiple tiny foci of non-specific white matter signal changes which were not in keeping with those seen in demyelinating conditions. Appointments with neurology and neuro-ophthalmology were expedited in view of worsening IR paresis and new-onset gaze-evoked nystagmus.

### Three weeks after presentation

**Figure 5:**
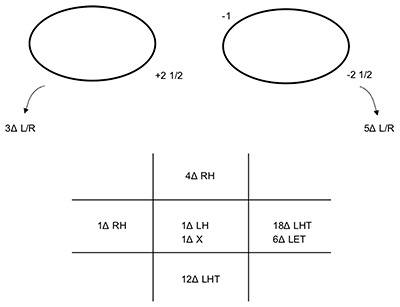
Ocular movements and prism cover test measurements (at 3 m) three weeks after presentation. Note: the arrows denote the BHTT measurements. LH: left hyperphoria, X: exophoria, LHT: left hypertropia, LET: left esotropia, RH: right hyperphoria.

**Figure 6:**
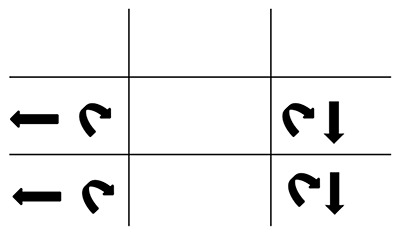
Nystagmus recording showing gaze-evoked mixed horizontal jerk and rotary nystagmus on dextro-version and -depression, which had a medium amplitude and moderate frequency. Also showing mixed rotary and downbeat nystagmus on laevo-version and laevo-depression, which had a medium amplitude and frequency.

**Figure 7:**
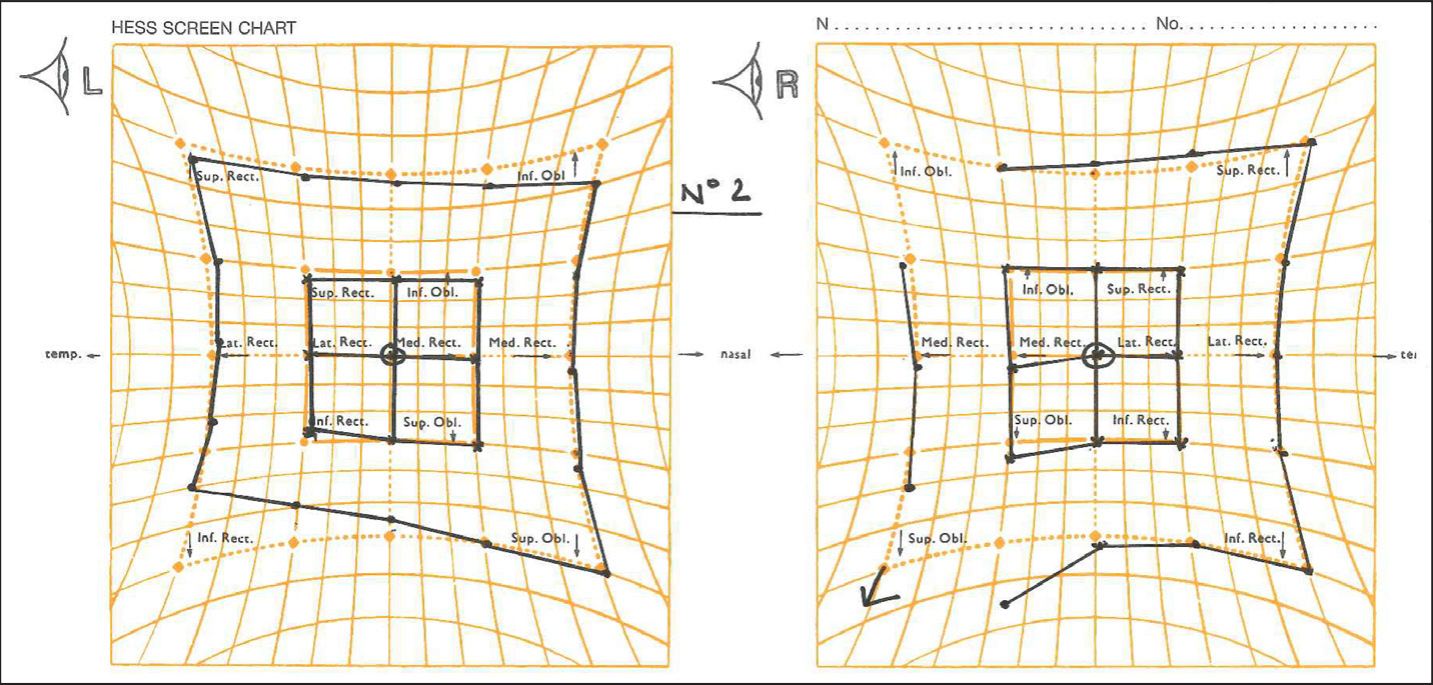
Hess chart showing that the muscle sequelae had now changed appearing to indicate a partial left third nerve palsy.

The patient returned to the orthoptic department for a joint appointment with the orthoptist and neuro-ophthalmologist. The patient advised that her neurologist had diagnosed her with stiff-person syndrome (SPS). This was based on typical symptoms, classic neuro-physiological changes, 10 white cells in the CSF, slightly raised CSF protein of 0.5, normal MRI imaging of the spine, some small white matter specks on the MRI brain and a GAD antibody level of >2000. She reported that she had received a course of intravenous immunoglobulin (IVIG) 4 days prior to her appointment and had been given diazepam 5 mg to take for the muscle spasms. VA right eye was 0.0 and left eye was 0.04 LogMAR. CT revealed a minimal left hyperphoria with minimal exophoria at near with intermittent diplopia and a minimal left hyperphoria with a minimal esophoria with intermittent diplopia in the distance. OM and PCT at 3 m measurements are shown in Figure 5. Diplopia was noted on laevo-version and laevo–depression, and the patient’s nystagmus had changed (Figure 6). Saccades remained unchanged. The Hess chart was suggestive of a partial left third nerve palsy (Figure 7). Despite the Hess chart appearing to suggest a partial third there were no signs of ptosis or pupil involvement. A decision was made to monitor the patient’s symptoms and review her in 3–4 weeks’ time.

### Six weeks after presentation

**Figure 8:**
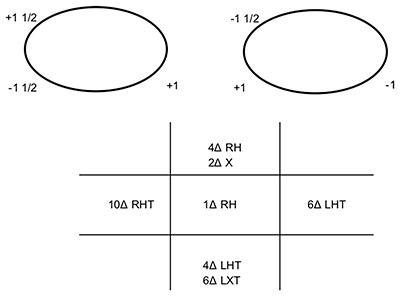
Ocular movements and prism cover test measurements (at 3 m) six weeks after presentation. RH: right hyperphoria, LHT: left hyertropia, LXT: left exotropia, RH: right hyperphoria, X: exophoria, RHT: right hypertropia.

**Figure 9:**
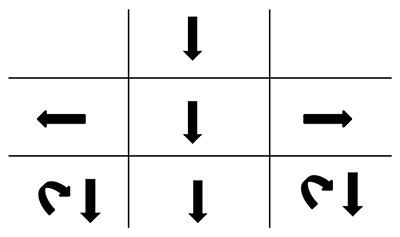
Nystagmus recording showing that the nystagmus had now progressed and primary positional downbeat nystagmus had developed which had a small amplitude and low frequency. The amplitude and frequency of the downbeat nystagmus was unchanged looking in elevation but increased when looking downwards. A right-beating jerk nystagmus was present on dextro-version and left-beating jerk nystagmus on laevo-version which had a medium amplitude and high frequency. On dextro-depression and laevo-depression a mixed rotary and downbeat nystagmus were present which had a small amplitude and high frequency.

**Figure 10:**
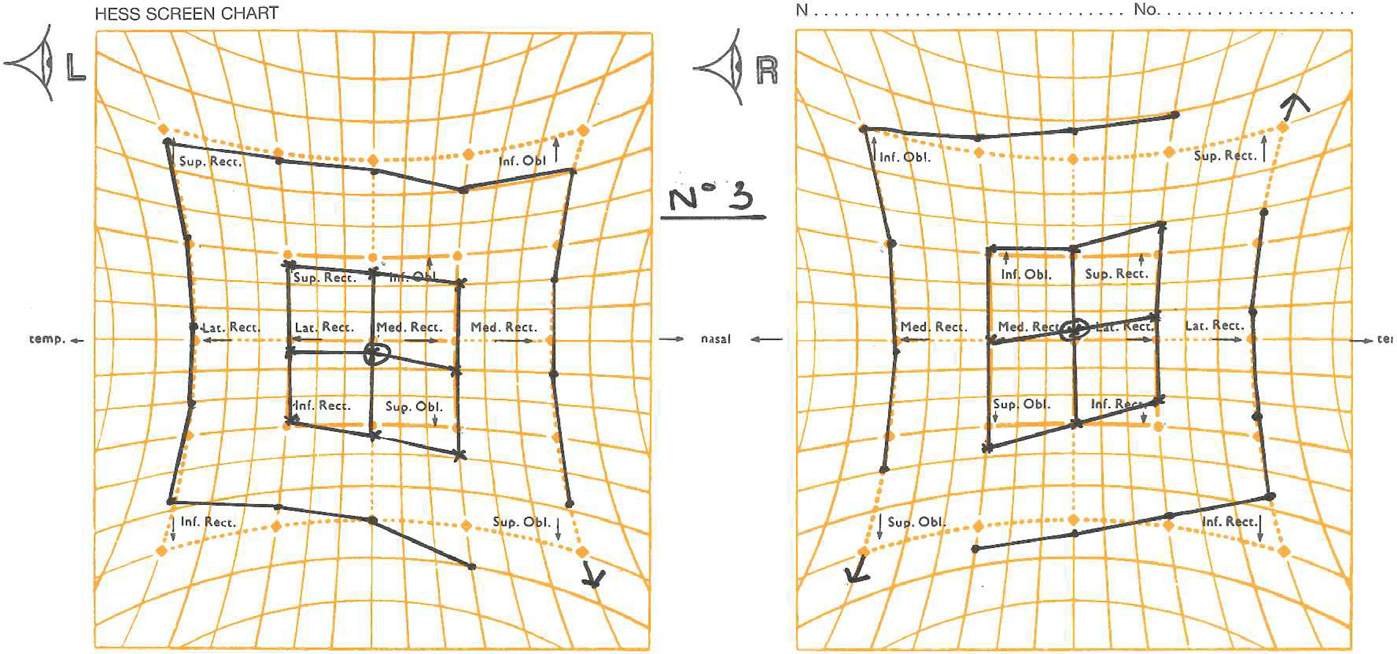
Hess chart now showing bilateral under-actions of the inferior rectus muscles. The left field still appears to indicate a partial third nerve palsy.

The patient reported that she was starting to be aware of the oscillopsia. She was happy as the IVIG and diazepam prescribed by her neurologist seemed to have relieved the muscle spasms she was having in her legs. Her neurologist, however, was concerned as her eye symptoms had remained unchanged despite having the IVIG. Due to the onset of disabling oscillopsia, the neurologist had prescribed gabapentin 100 mg 3× daily which was planned to be increased up to 300 mg 3× daily over a three-week period. VA right eye was 0.06 and left eye was 0.08 LogMAR. CT at near and distance noted a minimal right hyperphoria with diplopia prior to recovery. OM and PCT at 3 m measurements are shown in Figure 8. Diplopia was now noted on all dextro and laevo positions of gaze and in depression. The right inferior rectus now had a significant underaction, however, the left inferior rectus underaction appeared to have improved slightly. Nystagmus recordings are shown in Figure 9. The Hess chart appeared to suggest partial left third nerve palsy and right inferior rectus underaction (Figure 10). Still, despite the Hess appearing to suggest a partial left third nerve paresis there was no other evidence to support the diagnosis of third nerve palsy. It appeared that the patient had developed a laterally alternating skew deviation. The patient’s neurologist was considering plasmapheresis and steroids if the gabapentin did not help. The feeling of our specialist neuro-ophthalmologist was to try another IVIG; however, before this we needed to stop the diazepam. It was decided to review the patient again before deciding on the management plan.

### 9 weeks after presentation

The patient reported that she had now stopped taking the diazepam on advice from the consultant neuro-ophthalmologist and her muscle spasms had not returned. However, she complained of persistent diplopia and oscillopsia. The patient’s right inferior rectus weakness now also appeared to have become worse. The nystagmus was also now manifesting as oscillopsia in all gaze positions causing the patient much distress. The patient felt gabapentin was having no effect, so she decided to stop this despite advice that this may cause worsening of oscillopsia. A second IVIG was also being considered as the eye signs were not improving.

### 14 weeks after presentation

The patient returned for the joint orthoptic and ophthalmology follow-up appointment and reported that she had stopped gabapentin but then started to notice oscillopsia in primary position. She, therefore, restarted gabapentin 500 mg which had stopped the oscillopsia in primary position, however, she did not report a great deal of change to her eye movements overall and felt her eye symptoms were getting worse. The orthoptic investigation revealed no changes to her ocular status with the patient continuing to be binocular in primary position and manifest in all other gaze positions with nystagmus. It was decided at this visit that the patient would now have monthly IVIG for the foreseeable future until her ophthalmological problems were improved. Orthoptic management continues to be complete occlusion with Blenderm (3M™).

## Discussion

The patient discussed in this case report initially had a left inferior rectus weakness which developed into a laterally alternating skew deviation with nystagmus, and defective saccades. Further investigations highlighted no abnormalities. Blood tests for myasthenia gravis were negative and the patient’s MRI was normal except for numerous tiny foci of non-specific white matter signal changes. After a consultation with a neurologist, the patient was diagnosed with SPS. SPS is a rare neurological disorder which Dalakas (2008) has suggested a criteria for diagnosing (Table 1). However, patients not meeting the criteria may still be diagnosed with SPS, but are referred to as atypical (Dalakas 2008; Dalakas et al. 2000).

**Table 1:** Diagnostic criteria for SPS proposed by Dalakas (2008).


–	Muscular rigidity in the trunk and proximal limbs
–	Episodic muscle spasms, triggered by sounds, stress or touch
–	Continuous co-contraction of agonist and antagonist muscles, confirmed by electromyography
–	An absence of other neurologic disorders which cause stiffness and rigidity
–	Presence of serum anti-GAD antibodies
–	Good response to diazepa


There is currently a paucity of literature relating to ophthalmic problems patients with SPS experience and how they are best treated. A review of the literature has however revealed several case reports which have highlighted neuro-ophthalmic problems patients with SPS may present with (Chakravarthi, Goyal & Lal 2015; Economides & Horton 2005; Pittock, Lennon & McKeon 2009; Rothman et al. 2017; Thomas et al. 2005; Zivotofsky et al. 2006) and also those with only elevated levels of anti-glutamic acid decarboxylase antibodies (Ances et al. 2005; Antonini et al. 2003; Baizabal-Carvallo & Alonso-Juarez 2018; Brokalaki et al. 2015; Chen et al. 2015; Dubbioso et al. 2013; Farooq, Soin & Moss 2015; Tilikete et al. 2005) (Table 2). Nystagmus and defective saccades appear to be the most common presentation (Ances et al. 2005; Brokalaki et al. 2015; Chakravarthi, Goyal & Lal V 2015; Dubbioso et al. 2013; Economides & Horton 2005; Tilikete et al. 2005; Zivotofsky et al. 2006). Our patient had both of these problems. We postulate that the nystagmus experienced by our patient is most likely due to abnormal signals affecting the neural integrators within the brainstem and/or cerebellum. Downbeat nystagmus is typically due to disorders which affect the medulla and vestibulocerebellum. It is, therefore, possible that signals to such areas could have been affected, accounting for the primary positional nystagmus (Abadi 2002; Antonini et al. 2003; Yee 1989). Gaze-evoked nystagmus may be caused by a variety of medications and lesions. In particular, lesions affecting the nucleus prepositus hypoglossi and medial vestibular nucleus have caused gaze-evoked nystagmus (Abadi 2002). It is, therefore, also likely that these areas were affected in our patient.

**Table 2:** Reported neuro-ophthalmic problems associated with stiff-person syndrome and elevated levels of anti-glutamic acid decarboxylase antibodies.

Diagnosis	Neuro-ophthalmic associations	References

**Stiff-Person Syndrome**	Nystagmus – primary positional, endpoint, gaze-evoked	Economides & Horton 2005; Chakravarthi, Goyal & Lal V 2015; Pittock, Lennon & McKeon 2009
	Limited abduction	Chakravarthi, Goyal & Lal 2015; Pittock, Lennon & McKeon 2009
	Smooth pursuit deficits	Chakravarthi, Goyal & Lal 2015
	Saccadic deficits – initiation and accuracy	Economides & Horton 2005; Chakravarthi, Goyal & Lal 2015; Thomas et al. 2005; Pittock, Lennon & McKeon 2009; Zivotofsky et al. 2006
	Vertical and horizontal misalignment	Economides & Horton 2005; Chakravarthi, Goyal & Lal 2015; Nicholas et al. 1997; Saravanan, Paul & Sayeed 2002; Piccolo et al. 1990
	Tonic eye deviation	Economides & Horton 2005
	Myasthenia Gravis	Thomas et al. 2005, Nicholas et al. 1997; Saravanan, Paul & Sayeed 2002; Piccolo et al. 1990
	Thymoma	Thomas et al. 2005, Nicholas et al. 1997; Piccolo et al. 1990
	Supranuclear gaze palsy mimicking PSP	Pittock, Lennon & McKeon 2009
	Visual dysfunction and retinal pathology	Rothman et al. 2017

**Anti-Glutamic Acid Decarboxylase Antibodies**	Nystagmus – PAN, DBN, gaze-evoked	Ances 2005; Chen 2015
	Saccadic deficits – speed, accuracy and initiation	Dubbioso 2013
	Optokinetic nystagmus (OKN) deficits	Baizabal-Carvallo & Alonso-Juarez 2018
	Ocular flutter	Dubbioso 2013
	Square wave jerks	Baizabal-Carvallo & Alonso-Juarez 2018; Brokalaki 2015; Tilikete 2005
	Cerebellar ataxia	Dubbioso 2013
	Oculomotor dysfunction – including laterally abducting skew deviation	Ances 2005; Farooq 2015
	Epilepsy	Solimena 1988
	Cognitive dysfunction	Baizabal-Carvallo & Alonso-Juarez 2018; Tilikete 2005
	Vertigo	Antonini 2003

DBN – Downbeat Nystagmus, PSP – Progressive Supranuclear Palsy, PAN – Periodic Alternating Nystagmus.

Our patient also had a laterally alternating skew deviation. Interestingly, there have been two other similar reports. The first was a case from Ances et al. (2005), which reported the ophthalmic findings and response to treatment in a patient with glutamic-acid decarboxylase antibodies. The patient in their report also had downbeat nystagmus in primary gaze and an alternating skew deviation in lateral gaze positions. Their patient was very similar to our patient in the way they responded to treatment. The authors reported that diazepam improved the muscle spasms and rigidity, whereas, the IVIG treatment was only partially effective. Our patient’s muscle spasms were relieved within three-weeks of taking diazepam but no improvements in eye symptoms or ocular movements were seen after the first IVIG. However, other authors have reported good effects with IVIG (Chakravarthi, Goyal & Lal 2015; Farooq, Soin & Moss 2015; Pittock, Lennon & McKeon 2009; Rothman et al. 2017; Thomas et al. 2005; Zivotofsky et al. 2006).

The second case was published by Farooq et al. (2015). This report also had distinct similarities to our case. MRI imaging in their reported patient also showed subcortical white matter lesions which were also not consistent with demyelinating conditions such as multiple sclerosis. However, although the patient discussed in their paper had elevated levels of anti-GAD antibodies like our patient, they did not meet the diagnostic criteria required to be diagnosed with SPS. The authors suggested that the most likely cause of the laterally alternating skew deviation was related to cerebellar-brainstem changes associated with anti-GAD antibodies. Thus, it is sensible to postulate that these changes may have also caused the skew deviation seen in our patient.

Several reports have also established a connection between SPS and myasthenia gravis (Nicholas et al. 1997; Piccolo et al. 1990; Saravanan, Paul & Sayeed 2002; Thomas et al. 2005). Ocular motility findings in such patients have been motility restriction, defective saccades, and nystagmus. It is useful to, therefore, test patients with suspected SPS for the anti-ACH-R antibodies, as well as for anti-GAD antibodies. If suspicions of myasthenia are confirmed, then investigating patients for an enlarged thymus gland is indicated as thymectomy has been found to improve patients SPS and myasthenia symptoms (Thomas et al. 2005). Our patient did not test positively for myasthenia gravis, but the levels of the antibodies were at the high end of normal (our patient’s was 0.43 nmol/L with reference range: 0–0.44 nmol/L). It is therefore possible that our patient may go on to develop myasthenia gravis. Thomas et al. (2005) and Piccolo et al. (1990) reported patients developed myasthenia long after developing SPS.

As SPS is so rare, there is also a paucity of large evidenced-based trials looking at how best to treat patients, therefore, this means the quality of care for these patients is lacking compared to other similar conditions. The treatment given aims to relieve symptoms of stiffness/rigidity. Muscle relaxants drugs such as diazepam, gabapentin, baclofen, tiagabine, valproate, and carbamazepine are usually prescribed (Dalakas 2008; Hadavi et al. 2011; Lorish, Thorsteinsson & Howard 1989). These drugs help relieve the muscle spasms and hence the stiffness patients experience. For patients with persistent or severe symptoms, other treatments such as IVIG or plasmapheresis may be considered (Dalakas 2008; Dalakas et al. 2000; Hadavi et al. 2011). Orthoptic treatment may involve the use of prisms to re-establish binocular vision or occlusion in those unsuitable for prisms. Patients with stable and persistent ocular problems may be suitable for botulinum toxin injections and/or surgery. Drugs such as gabapentin, baclofen, and carbamazepine may also improve symptoms of troublesome oscillopsia (Dalakas 2008; Dalakas et al. 2000; Hadavi et al. 2011). Furthermore, the treatment of SPS requires good multi-disciplinary teamwork; continued communication was required between the orthoptists, ophthalmologists, and neurologists with the case described here. The neurologists relied heavily on the orthoptists feedback when deciding on whether to consider IVIG treatment in our case.

## Conclusion

This appears to be the third report of a laterally alternating skew deviation secondary to SPS and elevated levels of anti-GAD antibodies. We, therefore, suggest that SPS should be considered as a possible diagnosis in patients presenting with laterally alternating skew deviations or worsening cranial nerve palsies, nystagmus and muscle spasms with an unknown cause. Further research is required to develop an evidence-based approach to treating the ophthalmic issues patients with SPS experience. However, this may face difficulty due to the rarity of the condition. This case report highlights the importance and value of orthoptists and neuro-ophthalmologists in investigating and monitoring patients with stiff-person syndrome.
